# Little Patients, Big Issues: Something About Rapidly Growing Nodular Spitzoid Lesions in Childhood

**DOI:** 10.5826/dpc.1102a24

**Published:** 2021-03-08

**Authors:** Ilenia Marafioti, Maria Lentini, Carmelo Romeo, Serafinella P. Cannavò, Mario Vaccaro

**Affiliations:** 1 Department of Clinical and Experimental Medicine, Dermatology, University of Messina, Italy; 2 Department of Human Pathology of Adult and Childhood Gaetano Barresi, University of Messina, Italy

**Keywords:** Spitz nevus, spitzoid neoplasms, children, dermoscopy, surgery

## Introduction

Management of melanocytic lesions during childhood is often difficult. This is due to the rarity of melanoma during the first years of life and to rapid growth of lesions in younger patients regardless of their benign or malignant nature. Spitzoid lesions are very common in dermatological practice. This group of neoplasms includes Spitz nevi and spitzoid melanomas. Between these extremes there are atypical Spitz tumors of uncertain histological definition and malignant potential. Diagnosis is often controversial since they show clinical and biological variability, and no specific guidelines are available.

## Case Presentation

**Figure 1:**
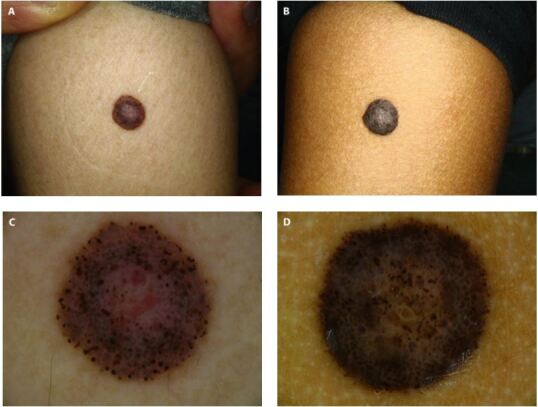
(A, B) Clinical and dermoscopic features of Spitz nevus at the beginning and (C, D) after 3 months: (A) a 6 × 6 mm roundish, unevenly brownish papule; (B) a central hypopigmented area with dotted vessels surrounded by inverse network associated with regularly distributed peripheral globules; (C) an 8 × 7 mm asymmetrical papule characterized by brown to dark intense pigmentation; and (D) a central blue-white veil and peripheral hyperpigmented network with pseudopods.

A 3-year-old healthy girl, with a family history positive for melanoma, presented with a roundish, unevenly pigmented, symptomless papule of recent onset on the lateral surface of her left leg (Figure 1A). Dermoscopy showed a central hypopigmented area with dotted vessels surrounded by inverse network associated with regularly distributed peripheral globules (Figure 1B). Clinical diagnosis was Spitz nevus, and the patient was scheduled for follow-up.

**Figure 2:**
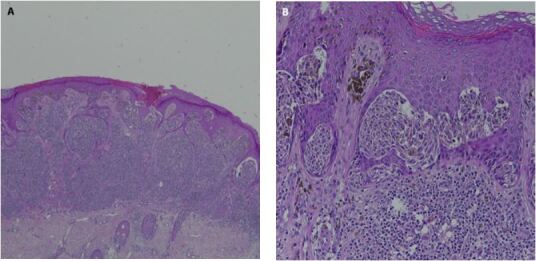
Histopathological examination revealed epidermal hyperplasia, lengthening of epidermal ridges, transepidermal elimination of moderately pigmented epithelioid melanocytic nests, and a lot of melanophages in the papillary dermis (H&E, original magnification [A] ×4 and [B] ×20×).

After 3 months, the lesion appeared nodular, asymmetrically enlarged and intensely pigmented (Figure 1C). Dermoscopy showed striking dermoscopic changes, namely, pseudopods with irregular distribution (peripheral and central), asymmetrically distributed globules of different size and color, and blue-white veil (Figure 1D). Despite the patient’s age, we had to remove the lesion. Histopathology revealed epidermal hyperplasia, lengthening of epidermal ridges, transepidermal elimination of moderately pigmented epithelioid melanocytic nests, and a lot of melanophages in the papillary dermis (Figure 2, A and B). A diagnosis of Spitz nevus without cytological atypia was made.

## Conclusions

Spitz/Reed nevi are acquired benign melanocytic lesions. They are mostly found on the face and lower limbs of children and young women [1]. They can be flat or nodular, hyper- or hypopigmented, and characterized by several dermoscopic patterns such as the starburst pattern (51%), regularly distributed dotted vessels (19%), and globular with reticular depigmentation (17%). Multicomponent, homogeneous, and reticular patterns can also be observed [2]. Histopathological examination may show epithelioid melanocytes (in childhood), spindle cells (in adolescence and adulthood), or both.

Spitz nevus is characterized by rapid growth, and sometimes both clinical history and dermoscopy may suggest spitzoid melanoma. From the data in literature we know that, though rare, pediatric melanoma has to be considered. According to Stefanaki et al. pediatric melanoma represents 1%–4% of all melanomas and 1%–3% of all pediatric malignancies, being the most frequent skin tumor in children. They distinguished 3 melanoma age groups, namely, neonatal (extremely rare), prepubescent children, and adolescents (having the same risk factors than adults). They also emphasized the importance of family history in children of all ages, because familial cases represent 5%–10% of melanomas [3].

Our excision could be considered an overtreatment, but this is very common in everyday dermatological practice. There are no standardized criteria or specific guidelines for management of spitzoid lesions, and their behavior can be tricky and unpredictable. In 2017, the International Dermoscopy Society proposed management guidelines in order to reduce unnecessary excisions without increasing the risk of underestimating misleading lesions. On the basis of the scheme, asymmetric Spitzoid tumors should always be excised, symmetrical nodular lesions should be excised or scheduled for close follow-up, while symmetrical flat lesions can be monitored until stabilization on the basis of patient’s age [2]. According to this algorithm, excision was unavoidable in our case. Nevertheless, dermoscopy is extremely important to select lesions that can be followed over time, such as our nevus at its onset.

Reports and in depth analysis of ambiguous lesions like this are essential to improve diagnostic accuracy and reduce surgery to bare minimum, especially when a little courage from dermatologists is required.
